# Sleep Disorders in Four Patients With Myotonic Dystrophy Type 1

**DOI:** 10.3389/fneur.2020.00012

**Published:** 2020-02-14

**Authors:** Yuka Urata, Masayuki Nakamura, Nari Shiokawa, Aiko Yasuniwa, Nagisa Takamori, Kensuke Imamura, Takehiro Hayashi, Takanori Ishizuka, Motofumi Kasugai, Akira Sano

**Affiliations:** Department of Psychiatry, Kagoshima University Graduate School of Medical and Dental Sciences, Kagoshima, Japan

**Keywords:** myotonic dystrophy type 1, excessive daytime sleepiness, orexin, sleep-onset rapid eye movement periods, frontal lobe dysfunction

## Abstract

Sleep disturbances such as excessive daytime sleepiness, central and obstructive sleep apneas, restless legs syndrome, and rapid eye movement sleep dysregulation are prominent in patients with myotonic dystrophy type 1 (DM1). Mild intellectual deficits presented in many patients with DM1. In addition, psychosocial issues caused by neuropsychiatric symptoms are a clinical problem. We herein present the cases of four DM1 patients with sleep disturbances and neuropsychiatric symptoms in the preceding stage of clinically significant muscle symptoms. One of the cases exhibited a sleep disorder and neuropsychiatric symptoms before electromyography showed myotonic discharge, suggesting that careful follow-up is also important. Patients 1 and 2 were first referred to our department due to daytime sleepiness. Patients 3 and 4 were objectively suffering from daytime sleepiness of which they were not subjectively aware of. Patients 1, 3, and 4 obtained high apnea–hypopnea index (AHI) scores, which reflected central and/or obstructive apnea, whereas patient 2 had an AHI score of zero. The daytime cerebrospinal fluid (CSF) orexin levels of all patients ranged from the normal lower limit to low, although they were not as low as those observed in narcolepsy with typical cataplexy. Neuropsychological tests of patients 1 and 2 showed frontal lobe dysfunction. Patients 3 and 4 were diagnosed with mild intellectual disability and autism spectrum disorder, respectively. All patients exhibited indifference toward their own symptoms, which may have resulted from the cognitive decline caused by DM1. Based on family history and/or neurological findings such as myotonia, we suspected DM1 as the cause of their sleep disturbances. Molecular analysis using the triplet repeat-primed polymerase chain reaction (TP PCR) method and Southern blotting, which provided a genetic confirmation of the diagnosis of DM1, were performed. These clinical features of sleep disturbances were unrelated to the length of CTG repeats and are caused by unknown molecular mechanisms. Clinicians should take into account that multisystem involvement in DM1 is hugely variable, and thus, a disabling sleep disorder could overshadow muscle impairment in DM1 patients.

## Background

Myotonic dystrophy type 1 (DM1; MIM 160900) is an autosomal-dominant disorder characterized by myotonia, muscular dystrophy, early-onset cataracts, endocrine abnormalities, and involvement of other organs, including the central nervous system (CNS) (1). DM1 is caused by a heterozygous trinucleotide repeat expansion (CTG)_n_ in the 3′ untranslated region (3′ UTR) of the dystrophia myotonica protein kinase gene on chromosome 19q13 (2). Sleep disturbances, including excessive daytime sleepiness (EDS), central and obstructive sleep apnea, restless legs syndrome, and rapid eye movement (REM) sleep dysregulation, are prominent in patients with DM1 (3, 4). The sleep problems associated with DM1 occasionally resemble those observed in narcolepsy, which is associated with decreased levels of orexin (hypocretin) in the cerebrospinal fluid (CSF). Several studies on DM1 patients with sleep disorders have been reported (3, 5–7); however, the pathogenesis remains unclear. Here, we report detailed clinical features of sleep disorders and neuropsychiatric symptoms preceding marked muscular symptoms in four patients with DM1.

## Case Presentation

### Patient 1

Patient 1 was a 26-year-old woman who was referred to the psychiatry department in our university hospital for evaluation of EDS. She presented with 15 years of EDS. Her history included falling asleep during a conversation while in a standing position. She also frequently caused traffic accidents by falling asleep while driving. She was admitted to our hospital under suspicion of narcoleptic sleep disorder.

She was obese (body mass index of 32.8 kg/m^2^; normal 18.5–24.9 kg/m^2^) and snored while sleeping. Regarding her past history, she had been treated for infantile enteritis and secondary amenorrhea. Her mother had been diagnosed with DM1. Neurological examination revealed that the patient had grip myotonia, which is known to be associated with DM1. In the laboratory examination, hypertriglyceridemia, hypercholesterolemia, and a decreased level of immunoglobulin G were found. Her intelligence quotient (IQ) score was measured during neuropsychological examination, using the Wechsler Adult Intelligence Scale III (WAIS-III). The patient's full-scale IQ (FIQ) was 87, her verbal IQ (VIQ) was 94, and her performance IQ (PIQ) was 82. Her frontal lobe function, as assessed based on the Frontal Assessment Battery (FAB), Trail Making Test, and Word Fluency Test, was not impaired; however, she failed the reverse digit span, suggesting that her attention was slightly limited. She also felt unconcerned about and indifferent toward any trouble she had caused, suggesting slight frontal lobe dysfunction.

She experienced EDS without sleep paralysis, cataplexy, or sleep-related hallucinations. Her score on the Epworth sleepiness scale (ESS) was 16; ESS scores range from 0 to 24, with scores 11–24 denoting increasing levels of EDS. Polysomnography (PSG) confirmed a diagnosis of severe central sleep apnea, with an elevated apnea–hypopnea index (AHI) of 40.2 (normal <5) events per hour and a 3% oxygen desaturation index (3% ODI) of 58.2 (normal <5) events per hour. Following the PSG, a multiple sleep latency test (MSLT) was performed to objectively quantify the degree of sleepiness. The results of the MSLT showed the following sleep latencies during five respective naps: 6, 3, 4, 3, and 6 min. The mean sleep latency was 4.4 min (normal ≥ 10 min). Three sleep-onset REM periods (SOREMPs; normal ≤ 1) were also noted. The HLA DQB1^*^0602 allele, which strongly supports a diagnosis of Japanese narcolepsy (8), was not detected. Her daytime CSF orexin level was 241 pg/ml (normal ≤ 200 pg/ml).

A diagnosis of DM1 was suspected, on the basis of her family history of DM1 and the symptom of grip myotonia. Electromyography was performed, and it showed myotonic discharges, which are well-known as a specific finding in DM1. We performed a molecular analysis to confirm DM1 using triplet repeat-primed polymerase chain reaction (TP PCR). The results of the TP PCR analysis revealed an expanded CTG repeat allele in the affected range (normal ≤ 35 repeats). Patient 1 was molecularly and clinically diagnosed with DM1, to which her sleep disturbance was attributed to.

### Patient 2

Patient 2 was a 32-year-old woman who had complained of EDS and fatigue since she was 20 years of age. She frequently had problems owing to falling asleep during meetings at work and while driving. For the purpose of assessing her for sleep disorders such as narcolepsy, she was referred to the psychiatry department and admitted to our hospital.

She had previously been given hormonal treatment for ovarian insufficiency. She had no relevant family history. In the neurological examination, she showed symptoms of grip myotonia and percussion myotonia. Atrophy of the sternocleidomastoid muscle was observed. She presented with bilateral juvenile cataracts. The data obtained from the laboratory examination were normal. Regarding the neuropsychological examinations, the WAIS-III revealed the following IQ scores: FIQ 73, VIQ 88, and PIQ 72. Assessments of frontal lobe function using the FAB, Modified Stroop Test, and Wisconsin Card Sorting Test revealed impairment of frontal lobe function. She had a diminished ability to concentrate, and her movement was slightly slower than normal.

She experienced EDS without sleep paralysis, cataplexy, or sleep-related hallucinations. Her ESS score was 19. Assessments for PSG were normal. In the subsequent MSLT, she fell asleep three times (sleep latencies of 5.1, 3.6, and 8.5 min) in five naps. Her mean sleep latency was 11.4 min, because the nap periods in which sleep did not occur were treated as 20-min latencies. Three SOREMPs were noted. The HLA DQB1^*^0602 allele was not detected. Her daytime CSF orexin level was 239 pg/ml.

Electromyography showed myotonic discharges. The results of the TP PCR analysis revealed an expanded CTG repeat allele. She was diagnosed with DM1, to which her sleep disturbance was attributed to.

### Patient 3

Patient 3 was a 23-year-old woman with a mild intellectual disability. She had been treated for pigeon toe up to the age of 3 years, as well as for at least 10 limb fractures, hyperopia, and amblyopia. Her father had been diagnosed with DM1 and died suddenly owing to an acute arrhythmia. She was admitted to our hospital for whole-body examination, with suspected DM1.

During the neurological examination, she exhibited grip myotonia, percussion myotonia, atrophy of the sternocleidomastoid muscle, and loss of grip strength. In the laboratory examination, elevated creatine kinase and a decreased level of immunoglobulin G were found. Her spirometry results showed restrictive pulmonary dysfunction. Distal muscle atrophy was seen on computed tomography. She was emotionally immature and had a general lack of understanding. We performed the WAIS-III for intelligence assessment, which revealed IQ values of FIQ 50, VIQ 54, and PIQ 57.

The mother of patient 3 had noticed that the patient snores and experiences sleep apnea; however, patient 3 was not aware of these problems herself. She had a score of 8 on the ESS. PSG confirmed the diagnosis of moderate obstructive sleep apnea, with an elevated AHI of 22.1 events per hour and a 3% ODI of 11.7 events per hour. In the subsequent MSLT, she fell asleep four times (sleep latencies of 20, 9.5, 13, and 15 min) in five naps. Her mean sleep latency was 15.5 min. Four SOREMPs were noted. Her daytime CSF orexin level was slightly low, at 180 pg/ml.

Electromyography showed myotonic discharges. Southern blot analysis revealed a CTG expansion of 1,300–1,500 repeats. The results of the TP PCR analysis revealed an expanded CTG repeat allele, confirming the diagnosis as DM1, to which her mild intellectual disability and sleep disturbance were attributed to.

### Patient 4

Patient 4 was a 27-year-old man with autism spectrum disorder (ASD). Regarding his past history, he had previously been treated for pediatric asthma. He was the older brother of patient 3. His father and sister (patient 3) had previously been diagnosed with DM1. He was also admitted to our hospital for the purpose of examination for DM1.

He had complained of numbness and pain in both lower limbs and a decline in grip strength. In the neurological examination, abnormalities such as myotonia and muscle atrophy were not noticeable. The data obtained from the laboratory examination were normal. The WAIS-III revealed IQ levels of FIQ 93, VIQ 92, and PIQ 97 and a significantly decreased processing speed index score in comparison with other indexes. Assessments of his development using the Parent Interview ASD Rating Scale and his development history confirmed the diagnosis of ASD. He had a relative lack of communication and social skills. He was unable to maintain work for prolonged periods.

The mother of patient 4 had noticed his EDS. He was indifferent to his sleep problems and had a score of six on the ESS. PSG confirmed a diagnosis of severe obstructive sleep apnea, with an elevated AHI of 30.5 events per hour and a 3% ODI of 18 events per hour. In the subsequent MSLTs, he fell asleep in all naps (sleep latencies of 14, 21, 14, 6, and 19.5 min). The mean sleep latency was 14.9 min. SOREMP was recorded only once. His daytime CSF orexin level was 228 pg/ml.

The results of a TP PCR revealed an expanded CTG repeat allele, confirming the diagnosis of DM1, to which his ASD and sleep disturbances were attributed to. Electromyography showed no abnormalities such as myotonic discharge, suggesting that the sleep disorder preceded the neuromuscular symptoms. Southern blot analysis revealed a CTG expansion of 250 repeats.

## Discussion

In this study, we presented detailed clinical features of sleep disorders in four patients with molecularly diagnosed DM1 (Table 1). Features of the sleep disorders in these patients were variable and non-specific. Patients 1 and 2 were first referred to the psychiatry department due to daytime sleepiness, which was reflected in their high ESS scores. Patients 3 and 4 were objectively suffering from daytime sleepiness of which they were not subjectively aware of, which was reflected in their low ESS scores. All patients exhibited an indifference toward their own symptoms, which may have resulted from the cognitive decline caused by DM1. Patients 1, 3, and 4 obtained high AHI scores, which reflected central and/or obstructive apnea, whereas patient 2 had an AHI score of zero. These differences of patients 1, 3, and 4 may be caused by altered central respiratory control and/or respiratory muscle weakness. REM sleep dysregulation is frequently observed in patients with DM1. Patients 1, 2, and 3 exhibited REM sleep dysregulation with three or four SOREMPs, whereas none were detected for patient 4. Patient 1 showed short sleep latency in MSLT, whereas patients 2, 3, and 4 did not. High-frequency SOREMPs and short sleep latency observed in patient 1 may be mainly caused by central sleep apnea with the highest AHI score. The daytime CSF orexin levels of all patients ranged from the normal lower limit to low, although they were not as low as those observed in narcolepsy with typical cataplexy (9). This low orexin level may partially cause EDS. The results of previous studies regarding CSF orexin levels in DM1 patients have been discrepant (5–7). These clinical features were unrelated to the length of CTG repeats and are caused by unknown molecular mechanisms. Generally, sleep disorders in DM1 are associated with a higher frequency of daytime sleepiness and REM sleep dysregulation (10); however, patients with DM1 may not exhibit distinctive symptoms at the individual level, as in this case series. Interestingly, one of the cases in this series had sleep disorder symptoms with severe obstructive sleep apnea before electromyography showed myotonic discharge, suggesting that careful follow-up is important.

**Table 1:** Clinical features of sleep disorders in four patients with DM1.

	**Sex and age at sampling**	**BMI (kg/m^**2**^)**	**CTG repeats**	**Myotonic discharge**	**ESS scores**	**Orexin (pg/ml)**	**AHI (number/h)**	**MSL (min)**	**SOREMPs (number)**	**Psychiatric symptom**
Normative values	NA	18.5–24.9	≤ 35	–	≤ 10	≤ 200	<5	≥10	≤ 1	–
Patient 1	F 26	32.8	≫130 TP PCR	+	16	241	40.2	4.4	3	Slight frontal lobe dysfunction
Patient 2	F 32	18.8	≫130 TP PCR	+	19	239	0	11.4	3	Frontal lobe dysfunction
Patient 3	F 23	17.2	1,300–1,550 Southern blotting	+	8	180	22.1	15.5	4	Mild intellectual disability
Patient 4	M 27	23.6	250 Southern blotting	–	6	228	30.5	14.9	1	Autism spectrum disorder

*BMI, body mass index; ESS, Epworth sleepiness scale; AHI, apnea–hypopnea index; MSL, mean sleep latency; SOREMPs, sleep-onset rapid eye movement periods; TP PCR, triplet repeat primed polymerase chain reaction; NA, not applicable; DM1, myotonic dystrophy type 1*.

## Conclusion

Clinicians should take into account that multisystem involvement in DM1 is hugely variable, and thus, a disabling sleep disorder could overshadow muscle impairment in DM1 patients.

## Ethics Statement

Genomic DNAs from peripheral blood samples were taken from all participants who gave written informed consent for DNA analysis and publication of this article. The research protocol and consent form were approved by the Institutional Review Boards of Kagoshima University. A copy of the written consent is available for review by the editor of this journal.

## Author Contributions

NS, AY, NT, and KI performed the treatment and neuropsychological tests and took the patient history. TH, TI, and MK supervised all steps of diagnoses and treatment. MN and AS supervised all steps of diagnoses and treatment and wrote the paper. YU summarized clinical features, performed genetic analysis, and wrote the paper.

### Conflict of Interest

The authors declare that the research was conducted in the absence of any commercial or financial relationships that could be construed as a potential conflict of interest.
